# Fear of dementia in adults without diagnosed dementia: an updated systematic review of prevalence, measurement, and correlates

**DOI:** 10.3389/fpubh.2026.1930480

**Published:** 2026-08-28

**Authors:** Feng Chen, Sixian Li, Kuai In Tam, Huimin Su

**Affiliations:** 1 Kiang Wu Nursing College of Macau, Macao, Macao SAR, China; 2 Nursing School of Hunan Normal University, Changsha, Hunan, China; 3 Department of Operating Room, Guangdong Provincial Key Laboratory of Major Obstetric Diseases, Guangdong Provincial Clinical Research Center for Obstetrics and Gynecology, The Third Affiliated Hospital, Guangzhou Medical University, Guangzhou, China

**Keywords:** adults, associated factors, cultural patterns, fear of dementia, systematic review

## Abstract

**Background:**

Dementia is a major public health concern. Adults, particularly older adults may experience fear of dementia due to concerns about current and future cognitive decline, loss of independence, or increased burden on their families. Previous reviews have mainly focused on concepts, measurement, or assessment tools. Therefore, it is necessary to summarize current evidence on fear of dementia among adults without a diagnosis of dementia.

**Objective:**

To primarily synthesize the reported prevalence or levels, measurement approaches, and associated factors of fear of dementia among adults without diagnosed dementia, and secondarily explore cultural patterns across Chinese/East Asian and Western contexts.

**Methods:**

Following PRISMA 2020 guidelines, eight Chinese and English databases were searched from inception to April 10, 2026. Study quality was assessed using JBI tools, and measurement properties were evaluated according to the COSMIN framework.

**Results:**

56 studies met the inclusion criteria. Study populations included adults aged 18 years or older, including university students, general adult populations, older adults living in the community, hospitalized older patients, and residents of long-term care facilities. Most standardized instruments demonstrated good internal consistency and structural validity; however, evidence remained insufficient regarding content validity, cross-cultural equivalence, measurement error, and responsiveness. Female, subjective memory complaints, depression, anxiety, negative attitudes toward aging, perceived personal risk of dementia, and a family history of dementia or previous contact with dementia were associated with higher levels of fear of dementia, whereas social support and favorable family functioning were associated with lower levels. The available evidence suggested that Western studies more often emphasized on the loss of autonomy, disruption of self-identity, and diminished perceived personal control, whereas patterns reported primarily in Chinese studies emphasized family burden, intergenerational responsibility, filial piety, and stigma. Evidence from other East Asian settings was insufficient to establish a broader regional pattern.

**Conclusion:**

Fear of dementia and related constructs were frequently reported across the included studies, although their prevalence and magnitude could not be estimated reliably because of substantial methodological and measurement heterogeneity. Future research should investigate whether incorporating its assessment into community-based cognitive health programs improves engagement, timely help-seeking, or psychological outcomes.

**Systematic review registration:**

https://www.crd.york.ac.uk/prospero/#searchadvanced, CRD420261419549.

## 1 Introduction

Dementia is a chronic neurodegenerative disease in later life, primarily characterized by progressive cognitive decline, impaired ability to perform activities of daily living, and behavioral and psychological symptoms (1). As population continues to age, the disease burden of dementia, family caregiving pressure, and socioeconomic impact are increasing (2). Beyond individuals diagnosed with dementia and their caregivers, dementia can also affect adults who have not received a diagnosis (3). In this review, adults without a formal diagnosis of dementia refer to individuals who have not been clinically diagnosed with dementia, including general adults, community-dwelling middle-aged and older adults, and people with subjective cognitive concerns, while excluding those with diagnosed dementia. These individuals may become concerned about developing dementia or specifically, Alzheimer’s disease in the future, especially for those after experiencing occasional forgetfulness, reduced attention, having relatives affected by the disease, or being exposed to dementia-related information (4, 5). Therefore, fear of dementia is not only individual psychological response, but is also closely related to cognitive health promotion, early screening, dementia risk counseling, and community-based public health management.

The literature uses several related but not fully equivalent terms, including fear of dementia, dementia worry, dementia-related anxiety, fear of Alzheimer’s disease, perceived threat of Alzheimer’s disease, and fear of memory loss (6). Fear of dementia mainly emphasizes an emotional response, whereas dementia worry primarily refers to cognitive concern, dementia-related anxiety may include emotional or physiological arousal, and perceived threat reflects perceived susceptibility to and severity of dementia. In this review, fear of dementia is used as an umbrella term to organize these related responses to the possibility of personally developing dementia or cognitive decline, rather than to suggest that these constructs or their measurement instruments are equivalent. Accordingly, “fear of dementia” is used throughout this review as the overarching term, whereas “dementia worry,” “dementia-related anxiety,” “fear of Alzheimer’s disease,” and other related terms are retained when describing the terminology, constructs, or instruments used in individual studies. It encompasses concerns about the future risk of developing dementia, loss of independence, disruption of self-identity, dependence on long-term care, increased family burden, and social evaluation. Moderate levels of fear of dementia may motivate individuals to pay greater attention to cognitive health, proactively participate in screening, and adopt health-promoting behaviors (7). However, excessive fear may lead to anxiety, sleep disturbances, catastrophic thinking, social withdrawal, and avoidance of medical care, thereby affecting the early identification and management of dementia risk (8). Understanding its levels, measurement approaches, and associated factors may support community screening, health education, dementia risk counseling, and psychological support.

Previous reviews have provided preliminary summaries of the concept, measurement, and associated factors of dementia worry, identifying several issues in the field, including inconsistent terminology, diverse measurement instruments, and insufficient theoretical grounding. In recent years, studies have also applied the Consensus-based Standards for the Selection of Health Measurement Instruments (COSMIN) methodology to evaluate the measurement properties of instruments assessing fear of dementia in older adults, thereby offering guidance for instrument selection. However, the existing literature still has several limitations. First, some previous reviews were published before recent Chinese-language studies, Asian studies, and newly developed or adapted instruments became available. Second, existing systematic reviews of measurement instruments have largely focused on older populations and scale properties, with limited efforts to simultaneously synthesize the prevalence, measurement approaches, and associated factors of fear of dementia (9, 10). Moreover, it remains unclear to what extent the reported prevalence or levels are comparable across studies using different constructs, instruments, and thresholds, and which associated factors show relatively consistent evidence across populations. Third, fear of dementia is not merely an individual psychological response but is also shaped by sociocultural context (11). Differences across cultural backgrounds in how people understand loss of autonomy, family responsibility, intergenerational caregiving, filial piety ethics, and stigma may lead to variation in the manifestations and influencing factors of fear of dementia. However, cultural patterns have rarely been critically synthesized, and observed differences may also reflect variation in study populations and measurement approaches. Based on these considerations, the primary objective of this updated systematic review was to synthesize the reported prevalence or levels, measurement approaches, and associated factors of fear of dementia among adults without a formal diagnosis of dementia. A secondary exploratory objective was to examine preliminary cultural patterns across Chinese/East Asian and Western contexts. The findings may clarify current evidence gaps and inform future research on community-based cognitive health screening, dementia risk counseling, public health education, early psychological support, and nursing intervention development.

## 2 Methods

### 2.1 Study design and registration

This review was undertaken as a systematic review and reported according to the PRISMA 2020 statement (12). The protocol was prospectively registered in PROSPERO (CRD420261419549).

### 2.2 Search strategy

We searched eight electronic databases, including PubMed, Web of Science, PsycINFO, the Cochrane Library, China National Knowledge Infrastructure (CNKI), Wanfang Database, Chinese Science and Technology Periodical Database (VIP), and Chinese Biomedical Literature Database (CBM). The search covered all records available from the establishment of each database to April 10, 2026. Search terms were organized around three key concepts: dementia or Alzheimer’s disease, cognitive decline or memory loss, and fear-, worry-, anxiety-, concern-, perceived threat-, or anticipatory dementia-related expressions. Database-specific free-text search strategies were adapted according to the search fields and functions available in each database. The PubMed search used proximity queries in the Title/Abstract field. For example, the complete PubMed search strategy was: (“dementia worry”[tiab:~4] OR “dementia worries”[tiab:~4] OR “dementia fear”[tiab:~4] OR “dementia fears”[tiab:~4] OR “dementia anxiety”[tiab:~4] OR “dementia concern”[tiab:~4] OR “dementia concerns”[tiab:~4] OR “dementia threat”[tiab:~4] OR “alzheimer worry”[tiab:~4] OR “alzheimer worries”[tiab:~4] OR “alzheimer fear”[tiab:~4] OR “alzheimer fears”[tiab:~4] OR “alzheimer anxiety”[tiab:~4] OR “alzheimer concern”[tiab:~4] OR “alzheimer concerns”[tiab:~4] OR “alzheimer threat”[tiab:~4] OR “anticipatory dementia”[tiab]). We also manually checked the reference lists of eligible studies and relevant reviews to identify additional studies. No additional eligible studies were identified through reference-list screening. The complete database-specific search strategies are presented in Supplementary Table 1.

### 2.3 Eligibility framework and inclusion/exclusion criteria

Articles were included if they met all of the following criteria:

1. The study population comprised adults aged 18 years or older who had not been diagnosed with dementia or severe cognitive impairment, including community-dwelling general populations, hospitalized individuals without dementia, residents of long-term care institutions.
2. Eligible studies assessed participants’ own future-oriented fear, worry, anxiety, perceived threat, or fear of memory loss regarding personally developing dementia, Alzheimer’s disease, or cognitive decline, based on item content rather than terminology alone.
3. The study was an original quantitative study, including cross-sectional studies, cohort studies, baseline data analyses of interventional studies, and scale development or psychometric validation studies.
4. The study reported at least one of the following outcomes: the prevalence, detection rate, level, or baseline score of fear of dementia or a related construct; the reliability, validity, or psychometric properties of instruments measuring fear of dementia; or factors associated with higher or lower levels of fear of dementia.
5. Master’s theses and doctoral dissertations were eligible if they reported original quantitative research, provided sufficient methodological and outcome data, and met the same population, construct, and study-design criteria as journal articles.
6. The full text was available.

Articles were excluded if they met any of the following criteria:

1. The study population consisted of individuals diagnosed with dementia or severe cognitive impairment, and data for participants without a diagnosis could not be extracted separately.
2. The study focused solely on caregiver burden, dementia-related stigma, clinical management of patients, or similar topics, without assessing participants’ own fear of dementia.
3. The study was purely qualitative research, a review, meta-analysis, conference abstract, commentary, editorial, expert opinion, study protocol, or letter.
4. Key data were missing and the relevant outcomes could not be extracted. When multiple reports described the same or a substantially overlapping study sample, they were treated as reports of a single study, and the report providing the most complete data relevant to the review outcomes was retained.

### 2.4 Study selection

Duplicate records were removed after the search results were imported into reference management software. Two reviewers independently screened the literature. Titles and abstracts were first reviewed to exclude records that clearly did not meet the inclusion criteria. Full texts were then assessed to further determine whether the study population, research topic, study design, and outcome indicators met the requirements of this review. During full-text assessment, eligibility was determined sequentially according to the population, measured construct, study design, relevant outcomes, and availability of extractable data. Each excluded full-text report was assigned one primary reason for exclusion. Any disagreements during the screening process were resolved through discussion between the two reviewers; if consensus could not be reached, a third reviewer made the final decision. The study selection process was presented using the PRISMA 2020 flow diagram (12).

### 2.5 Data extraction

Two reviewers independently extracted data using a predesigned form. Extracted information included author, year, country or region, study design, population, sample size, age characteristics, setting, terminology, measurement instrument, scoring method, prevalence or level of fear of dementia, associated factors, and psychometric properties. For scale development or validation studies, additional information was extracted on instrument structure, reliability, validity, cross-cultural validation, measurement error, and responsiveness. Discrepancies were resolved by discussion or consultation with a third reviewer. Study characteristics are presented in Supplementary Table 2.

### 2.6 Quality assessment

Quality assessment was conducted according to study type. Cross-sectional epidemiological studies were assessed using the JBI critical appraisal tool, focusing on sample representativeness, participant description, outcome measurement, confounding control, and statistical analysis (13). Scale development and validation studies were evaluated using the COSMIN Risk of Bias checklist, including content validity, structural validity, internal consistency, cross-cultural validity or measurement invariance, reliability, measurement error, criterion validity, hypothesis testing, and responsiveness (14). Quality assessment was used to interpret the credibility of evidence rather than to exclude studies. The JBI and COSMIN results are presented in Supplementary Tables 3, 4. Quality assessment informed the narrative interpretation: findings consistently reported in high-quality studies were considered more credible, whereas findings based mainly on moderate- or low-quality studies or measures with limited psychometric evidence were interpreted cautiously. Of the 56 included reports, 43 reports contributing cross-sectional epidemiological or baseline data were appraised using the JBI checklist, whereas 13 instrument-development, translation, or validation reports were evaluated according to the COSMIN framework and domains. Thus, all included reports were covered by one of the two appraisal approaches.

### 2.7 Data synthesis

The feasibility of meta-analysis was considered during evidence synthesis. However, because of substantial heterogeneity in study populations, cultural contexts, measurement instruments, scoring methods, outcome reporting, and analytical approaches, quantitative pooling was not performed. Only a limited number of studies used comparable construct definitions, instruments, scoring methods, thresholds, and reporting formats. Reported outcomes included general concern, severe fear, continuous scale scores, and moderate-to-high levels, while studies of associated factors also differed in effect measures, reference groups, and adjustment models. Therefore, descriptive synthesis and narrative analysis were used instead. Findings were summarized according to study characteristics, measurement approaches, prevalence or levels of fear of dementia, associated factors, and cultural patterns across Chinese/East Asian and Western contexts.

## 3 Results

### 3.1 Study selection results

**Figure 1:**
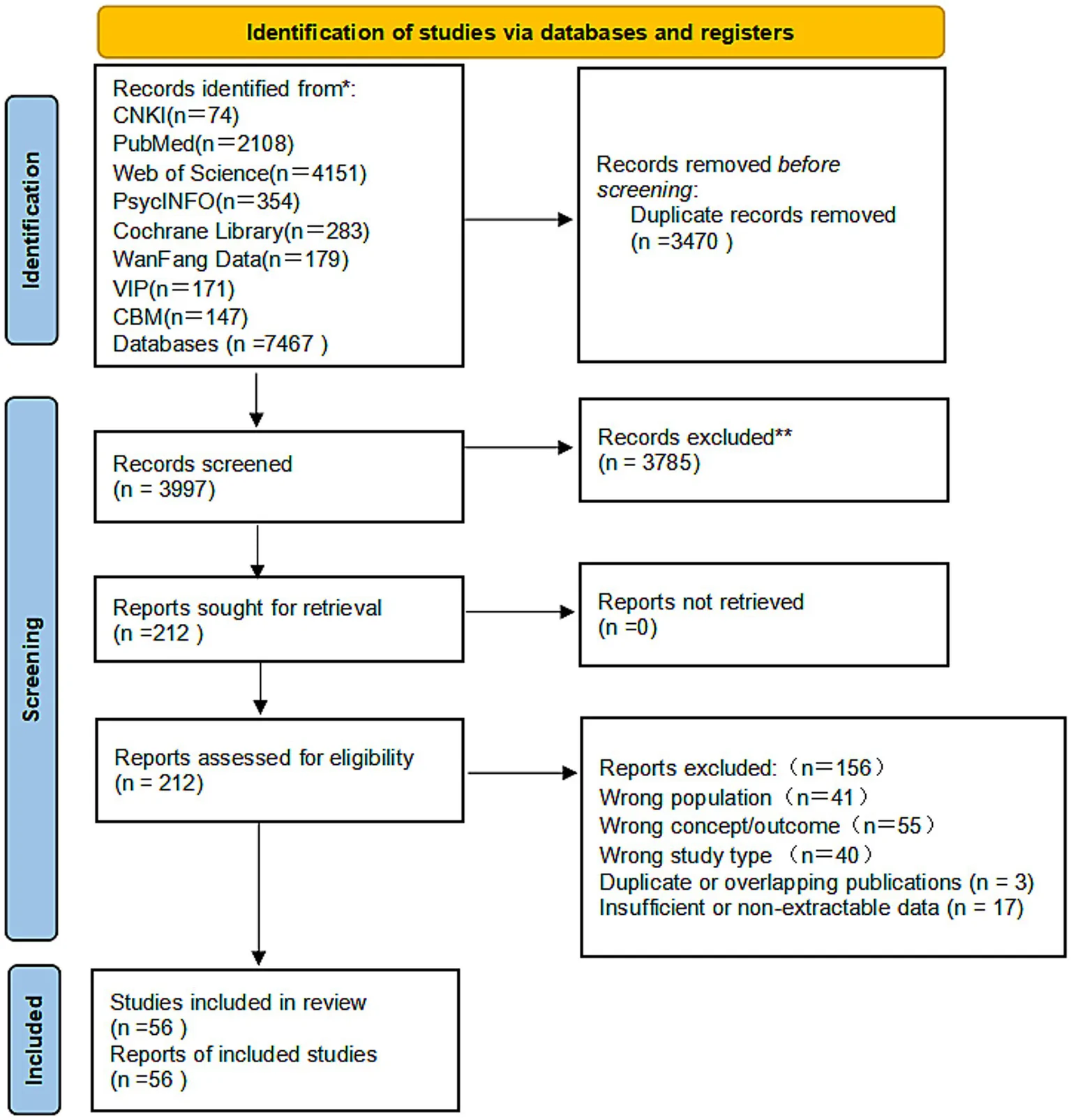
PRISMA flow diagram of the study selection process.

A total of 7,467 studies were identified through the systematic search. After removing 3,470 duplicate records, 3,997 records remained for title and abstract screening. Following the initial screening, 3,785 records were excluded, and 212 studies were sought for retrieval. All 212 reports were successfully retrieved and assessed for eligibility. Of these, 156 reports were excluded: 41 because the study population did not meet the inclusion criteria, 55 because the study concept or outcome indicators were not relevant to the review objectives, 40 because the study type did not meet the inclusion criteria, 3 because they were duplicate or overlapping publications, and 17 because relevant data were insufficient or could not be extracted. Finally, 56 studies, corresponding to 56 reports, were included in the review, comprising 42 English-language articles and 14 Chinese-language articles (6–8, 11, 15–66). The study selection process is shown in Figure 1.

### 3.2 Basic characteristics of the included studies

Among the 56 original quantitative studies, 42 were English-language articles and 14 were Chinese-language articles (16–29). The studies were conducted across Asia, Europe, North America, and the Middle East (35), including mainland China and Hong Kong (7, 16–29, 63, 65), Japan (50, 57), South Korea (11, 46, 49, 51, 52, 66), the United States (6, 31, 33, 34, 36, 38, 45, 53, 55, 58), the United Kingdom (47, 48, 64), Germany (37, 39, 54, 55), and Israel (32, 62). Most studies used cross-sectional designs, while several were scale development or validation studies (8, 15, 28, 30, 40, 41, 56, 58, 66). Study populations included general adults (33, 36, 39, 45, 47, 48, 55, 62, 64), community-dwelling middle-aged and older adults (24, 26, 27), hospitalized older patients (19, 29), older adults in long-term care institutions (24, 26, 27), and university students or young adults (36, 38, 45).

Measurement approaches varied across studies. Commonly used tools included the Fear of Dementia Scale (FODS), the Fear of Alzheimer’s Disease Scale (FADS), and their adapted versions (6, 7, 16, 17, 20, 21, 28–30, 40, 42, 43, 46, 47, 52, 53, 58, 63, 65, 66); the Dementia Worry Scale (DWS), the Modified Dementia Worry Scale (MDWS), and their adapted versions (4, 15, 31, 36, 37, 44, 45, 53); single-item self-report questions (32, 34, 55, 57, 61); the Visual Analogue Scale (VAS) or Numerical Rating Scale (NRS) (5, 51); and newer instruments such as the Fear and Avoidance of Memory Loss Scale (FAM) (8, 56) and the Threat of Dementia Scale (ToDS) (41). Reported outcomes included prevalence estimates, detection rates, fear or worry scores, stratified levels, associated factors, and psychometric properties. Overall, the included studies showed considerable heterogeneity in study populations, terminology, measurement instruments, scoring methods, and outcome reporting formats. A summary of the basic characteristics is provided in Table 1, detailed study-level information is presented in Supplementary Table 2, and detailed psychometric properties are summarized in Supplementary Table 5.

**Table 1:** Summary of the basic characteristics of the included studies.

Item	Summary
Number of included studies	56 original quantitative studies
Language	42 English-language articles and 14 Chinese-language articles (16–29)
Study design	Most studies were cross-sectional studies, with additional scale development and validation studies.
Study regions	China, Japan, South Korea, the United States, the United Kingdom, Germany, Israel, the Middle East, and other regions
Study populations	General adults, community-dwelling middle-aged and older adults, hospitalized older patients, older adults residing in long-term care institutions, and young populations
Core terminology	Fear of dementia, dementia worry, dementia-related anxiety, fear of Alzheimer’s disease, fear of memory loss, and related terms
Measurement approaches	Single-item questions, NRS/VAS, self-developed questionnaires, FADS, FODS, DWS/MDWS, FAM, ToDS, and other instruments
Main outcomes	Fear of dementia scores, proportions reporting worry, detection rates of moderate-to-high levels, associated factors, and psychometric properties of measurement instruments

### 3.3 Methodological quality of the included studies

The JBI appraisal was conducted for 43 cross-sectional epidemiological studies (13). Overall, 35 studies (81.4%) were rated as high quality, 5 (11.6%) as moderate quality, and 3 (7.0%) as low quality. Most studies clearly defined the inclusion criteria, described the participants and settings, and used appropriate statistical analyses. The main limitations concerned unclear reporting of measurement validity, sampling frame, sample size estimation, non-response, and the identification or control of confounding factors. Detailed study-level JBI appraisal results are presented in Supplementary Table 3.

For scale development, revision, and validation studies, the COSMIN-based evaluation showed that most standardized instruments reported internal consistency and structural validity, mainly supported by exploratory factor analysis, principal component analysis, or confirmatory factor analysis (14). Translation and cultural adaptation procedures were described for several Chinese and Korean versions, and test–retest reliability was reported for selected instruments. However, evidence for content validity, cross-cultural validity or measurement invariance, measurement error, criterion validity, and responsiveness was limited or absent. Detailed COSMIN-domain evidence and numerical psychometric properties are provided in Supplementary Tables 4, 5.

Quality-informed interpretation indicated that the strength of support varied across the principal findings. Evidence concerning prevalence and subjective cognitive concerns was methodologically mixed and heterogeneous. The association of anxiety or depression with higher fear of dementia was supported predominantly by high-quality studies. Social support or favorable family functioning was associated with lower fear of dementia in two high-quality studies, but the small evidence base limited confidence in this finding. Although several studies informing the cultural interpretation received favorable ratings, the evidence was based on indirect and geographically imbalanced comparisons and was therefore interpreted cautiously.

### 3.4 Measurement approaches for fear of dementia

Across the included studies, measurement approaches for fear of dementia could be classified into three broad categories (10). The first category comprised single-item or brief questions, which were commonly used in large population surveys to assess whether respondents were worried about developing dementia or the degree of such worry (32, 34, 55, 57, 61). The second category included numeric rating scales, visual analogue scales, and self-developed questionnaires, which were used to rapidly assess the intensity of dementia-related worry or perceived disease risk (5, 39, 51). Although these brief approaches are easy to administer and reduce respondent burden, they have limited ability to capture the multidimensional nature of fear of dementia, and their comparability and psychometric evidence are often limited.

The third category comprised standardized multidimensional instruments, including the FADS, FODS, DWS/MDWS, FAM, and ToDS (10). The FADS focuses on Alzheimer’s disease-specific fear and has been validated or adapted in Korean and Chinese populations (6, 30, 58, 66). The FODS assesses broader fear of dementia and has been applied in community and long-term care settings (16, 28, 40, 42). The DWS/MDWS mainly measures persistent dementia-related worry (15, 45), whereas the FAM incorporates both fear of memory loss and avoidance behavior (8, 18, 56). The ToDS emphasizes the perceived threat of dementia to self-identity, dignity, and sense of existence (41). The main measurement instruments and their characteristics are summarized in Table 2.

**Table 2:** Summary of representative measurement instruments and applicable contexts.

Instrument type	Representative instruments	Core measurement content	Strengths	Limitations	Applicable contexts
Single-item or brief questions	Single-item self-report questions, NRS, VAS	Intensity of worry or fear	Simple and suitable for large-sample surveys	Unable to capture multidimensional structure; limited reliability and validity evidence	Population monitoring and rapid screening
Alzheimer’s disease fear instruments	FADS and its Korean and Chinese adaptations	General fear, physical symptoms, catastrophic attitudes	Relatively comprehensive structure and widely applied	Relatively large number of items; insufficient content validity in some versions	Assessment of Alzheimer’s disease-specific fear
Fear of dementia instruments	FODS, Chinese version of the FODS	Cognitive, social, physiological, and related dimensions	Suitable for assessing broader fear of dementia; frequently used in Chinese studies	Insufficient evidence regarding measurement error and responsiveness	Screening among community-dwelling and clinical older adults
Dementia worry instruments	DWS, MDWS	Persistent worry about developing dementia in the future	Fewer items and suitable for survey research	More focused on “worry,” with limited dimensional coverage	Cross-sectional surveys and longitudinal follow-up
Fear and avoidance of memory loss instruments	FAM, Chinese version of the FAM	Fear of memory loss and avoidance behavior	Adds the behavioral dimension of “fear–avoidance”	Cross-cultural validation still needs strengthening	Studies of medical care avoidance and screening avoidance
Dementia threat instruments	ToDS	Threats to self-identity, dignity, and sense of existence	Emphasizes psychological existential threat	Limited applied research	Geriatric mental health research

NRS, numeric rating scale; VAS, visual analogue scale; FADS, Fear of Alzheimer’s Disease Scale; K-FADS, Korean version of the Fear of Alzheimer’s Disease Scale; FODS, Fear of Dementia Scale; DWS, Dementia Worry Scale; MDWS, Modified Dementia Worry Scale; FAM, Fear and Avoidance of Memory Loss Scale; ToDS, Threat of Dementia Scale.

Overall, brief measures were used mainly in large population surveys and commonly produced categorical estimates of concern or fear, whereas standardized multidimensional instruments were more often used to assess continuous scores and specific domains, such as persistent worry, physical symptoms, catastrophic attitudes, avoidance, and threats to self-identity (6, 8, 34, 40, 41, 54, 57). Multidimensional instruments provided broader construct coverage and generally more extensive psychometric evidence; however, differences in their conceptual focus, scale structure, and cultural adaptation limited direct comparisons across studies (Table 2; Supplementary Tables 4, 5).

### 3.5 Reported proportions and levels of fear of dementia

Fear of dementia and related constructs were reported across diverse adult populations without a formal diagnosis of dementia. However, their prevalence and magnitude could not be estimated reliably because the included studies used non-equivalent constructs, instruments, thresholds, populations, and reporting formats. Owing to differences in measurement instruments and reporting formats, some studies reported proportions of participants with dementia-related concern, marked worry, severe fear, or moderate-to-high levels of fear, whereas others reported mean scores, medians, or stratified levels. Some studies also used latent class analysis or latent profile analysis to identify different patterns of fear of dementia (20). Accordingly, the range of 12.7–68.0% should be interpreted as a summary of heterogeneous study-specific proportional outcomes rather than as a directly comparable prevalence range. Among studies reporting proportional outcomes, the reported proportions ranged from 12.7 to 68.0%. The lowest estimate (12.7%) represented severe fear assessed using a single-item question among German adults aged 40–70 years (54), whereas the highest estimate (68.0%) represented moderate-to-high FODS levels among older adults in long-term care institutions in China (26). These estimates were based on different constructs, instruments, thresholds, and populations and should not be interpreted as directly comparable prevalence estimates (Supplementary Table 2).

The variation in reported prevalence was closely related to measurement approach and study population. Single-item questions usually captured general concern or severe fear, whereas standardized multidimensional instruments more often reported continuous scores or categorical levels. Therefore, direct numerical comparisons across studies should be interpreted with caution. Higher levels of fear were more commonly reported among older adults in long-term care institutions, hospitalized older patients, rural older adults, individuals with subjective memory complaints, and those with a family history of dementia or prior exposure to dementia (59). Middle-aged and young adults could also experience dementia-related worry, particularly when they had dementia-related exposure, family history, or self-perceived cognitive decline. Importantly, favorable JBI ratings for several individual studies did not overcome the lack of comparability among the reported proportions, and confidence in any overall prevalence estimate therefore remained limited.

### 3.6 Factors associated with fear of dementia

The factors associated with fear of dementia were grouped into four domains: sociodemographic factors, health- and cognition-related perception factors, psychological factors, and social environmental factors and exposure history (7, 31–33, 35, 36, 65).

#### 3.6.1 Sociodemographic factors

Women were more likely than men to report higher levels of fear of dementia (10, 23, 54, 55, 63). The relationship between age and fear of dementia was inconsistent. Some studies suggested that fear levels were higher among middle-aged or young-old adults, whereas fear may decline in advanced old age, showing a nonlinear or mixed pattern (11, 37, 54). Findings regarding education, marital status, and living arrangement were also mixed. However, individuals living alone, widowed individuals, or those with limited perceived support tended to report higher levels of fear (10, 23–25, 39, 49, 63). The associations involving anxiety or depression were supported predominantly by high-quality studies and were therefore considered relatively more consistent in direction. Evidence concerning subjective cognitive concerns was derived from studies of mixed quality, whereas the social-support finding was based on only two high-quality studies and was therefore regarded as more limited.

#### 3.6.2 Health- and cognition-related perception factors

Subjective memory complaints or perceived cognitive decline were among the most consistently reported positive correlates of fear of dementia (32, 34, 44, 53). Poorer self-rated health, chronic disease burden, and sleep problems were also associated with higher levels of fear (49, 50, 54, 65). The relationship between dementia knowledge or literacy and fear was inconsistent. Some studies suggested that greater knowledge may reduce misconceptions, whereas others found that higher knowledge was associated with stronger perceived threat or worry (43, 59, 64).

#### 3.6.3 Psychological factors

Depression, anxiety, trait anxiety, negative attitudes toward aging, and higher perceived personal risk of dementia were generally associated with higher levels of fear of dementia (10, 21, 31, 36, 37, 50, 63, 64).

#### 3.6.4 Social environmental factors and exposure history

A family history of dementia, caregiving experience, or close contact with people living with dementia was generally associated with higher levels of fear (25, 44, 50, 61). In contrast, social support and favorable family functioning were generally associated with lower levels of dementia-related fear or worry (11, 23). Stigma, perceived social threat, and concerns about becoming a burden to the family were also reported as relevant social-contextual factors (31, 41). Overall, subjective memory complaints or perceived cognitive decline (32, 34, 44) and depression- or anxiety-related variables (37, 50) showed the most consistent positive associations with fear of dementia. Negative attitudes toward aging and self-perceived dementia risk (10, 21, 64), as well as a family history of or personal exposure to dementia (25, 44, 50), also generally showed positive associations, whereas findings for age, education, and dementia knowledge or literacy were mixed (11, 37, 54, 64). The evidence on associated factors is summarized in Table 3.

**Table 3:** Summary of evidence on factors associated with fear of dementia.

Domain	Specific factor	Direction	Summary of evidence	Representative evidence
Sociodemographic factors	Gender	↑ in women	Women may report higher fear of dementia, possibly reflecting caregiving expectations, health anxiety, and perceived family responsibility.	Werner et al. (10); Meyer et al. (54); Wu et al. (63); Hajek and Koenig (55)
	Age	non-linear/mixed	fear of dementia may be higher in midlife or early old age and may decline in later old age; findings vary across samples and measures.	Ryu and Park (11); Meyer et al. (54); Bowen et al. (37)
	Education	±	Evidence is mixed. Education may increase dementia literacy and risk awareness, but its association with fear/worry depends on context, measurement, and sample characteristics.	Hajek and Koenig (39); Joo et al. (49); Wu et al. (63)
	Living alone, widowhood, or being unmarried	↑	Lower perceived availability of future informal care and social support may increase uncertainty about dementia-related dependency.	Werner et al. (10); Li and Li (24); Ran and Zhang (25)
Health and perceived cognition	Subjective memory complaints/perceived cognitive decline	↑	Subjective memory problems may trigger catastrophic interpretations of everyday cognitive lapses and anticipatory anxiety about dementia.	Werner (32); Cutler and Hodgson (34); Lee et al. (44)
	Chronic disease or poor self-rated health	↑	Poorer perceived health may heighten vulnerability beliefs and perceived disease threat.	Joo et al. (49); Nakayama et al. (50); Meyer et al. (54)
	Sleep problems/insomnia	↑	Sleep problems may interact with anxiety, stress, and cognitive concerns, thereby reinforcing dementia-related fear.	Joo et al. (49); Dai et al. (65)
	Dementia knowledge/literacy	±	A possible “knowledge paradox” may exist: greater knowledge can improve awareness but may also increase perceived threat in some groups.	Ebert et al. (43); Liu et al. (59); Ashworth et al. (64)
Psychological factors	Depression, anxiety, and trait anxiety	↑	Negative affect may increase attention to memory lapses and perceived risk of future cognitive decline.	Bowen et al. (37); Nakayama et al. (50)
	Negative attitudes toward aging/ageism	↑	Viewing aging as synonymous with dependency or cognitive decline may amplify dementia-related fear.	Werner et al. (10); Tan et al. (21); Wu et al. (63)
	Self-perceived dementia risk	↑	Higher perceived susceptibility is consistently linked to stronger perceived threat of developing dementia.	Maxfield and Greenberg1 (31); Ashworth et al. (64)
Social environment and exposure history	Family history of dementia or personal exposure to dementia	↑	Direct or family exposure may increase perceived severity, familiarity with dependency, and perceived uncontrollability of dementia.	Ran and Zhang (25); Lee et al. (44); Nakayama et al. (50)
	Social support/family functioning	↓	Supportive social and family resources may buffer concerns about future care needs, dependency, and loss of independence.	Ryu and Park (11); Wu et al. (23)
	Stigma/perceived social threat	↑	Fear of labeling, social exclusion, and becoming a family burden may intensify fear of dementia.	Maxfield and Greenberg (31); Cheston et al. (41)

↑ indicates an association with higher fear of dementia; ↓ indicates an association with lower fear of dementia; ± indicates mixed or inconsistent evidence.

### 3.7 Preliminary cultural patterns across Chinese, other East Asian, and Western contexts

The included studies suggested that fear of dementia may be shaped by cultural context, although the available evidence should be interpreted as evidence-informed patterns rather than definitive cross-cultural differences (10, 62). In Western studies, fear of dementia was more often measured and interpreted in relation to loss of autonomy, impaired cognitive functioning, threats to self-identity, loss of control, and existential threat. Related instruments frequently included content on “loss of self,” “loss of independence,” and perceived threat to personal identity or dignity (6, 36, 41, 42, 53).

In studies conducted primarily in China, fear of dementia was more commonly discussed in relation to family responsibility, intergenerational relationships, caregiving burden, filial piety, stigma, and concerns about becoming a burden to family members (21, 23, 59, 63). Limited related evidence was identified in one South Korean study (11), whereas the included Japanese studies did not directly address these specific themes. Evidence was therefore insufficient to establish a broader East Asian regional pattern. However, these observed differences may also reflect variation in research questions, sample sources, and instrument selection. For example, Western studies more often used instruments emphasizing self-identity or existential threat, whereas primarily Chinese studies more frequently included community-dwelling, rural, hospitalized, or long-term care older adults and examined family or social support variables (17, 19, 24, 26, 29, 63). Therefore, because these observations were derived from indirect comparisons across studies rather than direct cross-cultural comparisons, they should be regarded as preliminary and hypothesis-generating rather than confirmed cultural differences.

## 4 Discussion

This review included 56 quantitative studies and synthesized evidence on the prevalence, measurement approaches, associated factors, and cultural contexts of fear of dementia among adults without a formal diagnosis of dementia (6–8, 11, 15–66). Across the included studies, fear of dementia and related constructs were frequently reported in diverse adult populations. However, their prevalence and magnitude remain uncertain because of substantial heterogeneity in constructs, instruments, thresholds, populations, and reporting formats. However, confidence in the magnitude of this finding is limited by the predominance of cross-sectional designs, substantial heterogeneity, and incomplete evidence for several measurement properties (Supplementary Tables 3, 4). This variation may be related to differences in study populations, measurement instruments, scoring methods, cut-off criteria, and cultural contexts (10). Compared with previous reviews that focused mainly on concepts, measurement instruments, or older adults, this review incorporated both Chinese- and English-language studies and populations across adulthood, providing a broader evidence map of fear of dementia as a psychosocial phenomenon (9, 10).

### 4.1 Explaining heterogeneity in the prevalence of fear of dementia

This review found substantial variation in study-specific proportions of marked worry, severe fear, or moderate-to-high levels of fear of dementia, which ranged from approximately 12.7 to 68.0%. These outcomes were not estimates of an equivalent construct and should not be interpreted as a directly comparable prevalence range. This variation may first be explained by differences in study populations. Older adults residing in long-term care institutions, hospitalized older patients, rural older adults, individuals with subjective memory complaints, and those with a family history of dementia or caregiving-related exposure may perceive dementia as a more personally relevant threat and therefore report higher levels of fear (20, 59, 61). By contrast, dementia-related worry among general community-dwelling adults or young populations may be more strongly influenced by media information, relatives’ illness experiences, or self-perceived cognitive changes.

Differences in measurement instruments and cut-off criteria also contributed to heterogeneity. Some studies used single-item questions or numeric rating scales, whereas others used standardized instruments such as the FADS, FODS, DWS/MDWS, FAM, or ToDS, which assess different dimensions, including fear, worry, avoidance, somatic responses, and threats to self-identity (6, 8, 15, 40–42, 58, 62). Even when the same instrument was used, studies differed in their use of mean scores, medians, latent classes, or proportions with moderate-to-high levels. Therefore, prevalence estimates should not be directly compared without considering the measurement instrument, study population, scoring method, and cultural context.

### 4.2 Measurement issues and future directions for instrument development

Terminology remains inconsistent in this field. Although terms such as fear of dementia, dementia worry, dementia-related anxiety, fear of Alzheimer’s disease, perceived threat of Alzheimer’s disease, and fear of memory loss all refer to negative expectations about future cognitive decline, their measurement emphases differ (6). For example, the FADS focuses more on Alzheimer’s disease-specific fear and catastrophic attitudes, the FODS on broader dementia fear, the DWS/MDWS on persistent worry, the FAM on fear and avoidance of memory loss, and the ToDS on threats to self-identity and sense of existence (6, 8, 15, 40–42, 58, 62).

The COSMIN assessment indicated that most standardized instruments reported internal consistency and structural validity. However, evidence for content validity, cross-cultural measurement invariance, measurement error, and responsiveness remains limited (14, 30). This suggests that existing tools may be suitable for population surveys and preliminary screening, but their use in individual clinical decision-making, intervention evaluation, or cross-cultural comparison requires caution. Future research should prioritize the refinement of existing instruments based on clearer construct definitions, target-population interviews, cognitive interviews, and cross-cultural validation, rather than simply developing new tools (9, 10, 15, 30, 66).

Beyond measurement differences, heterogeneity may also reflect variation in participant age, population type, and country of study. Studies of students or younger adults may capture anticipatory concerns (36, 41, 61), whereas studies of community-dwelling, hospitalized, or institutionalized older adults may reflect concerns related to cognitive decline, health status, and dependency (19, 24, 26, 27, 29). Apparent country-level variation may additionally reflect differences in cultural and healthcare contexts, although direct comparative evidence remains limited (11, 21, 23, 59, 63).

### 4.3 Associated factors and potential mechanisms

The observed associations may be interpreted through the Health Belief Model and the fear–avoidance mechanism (31, 46). Fear may be particularly pronounced when individuals perceive themselves as highly susceptible to dementia, view the disease as severe and uncontrollable, and believe that their coping resources are insufficient. Subjective memory complaints, family history, and prior exposure to dementia may also heighten perceived susceptibility and severity, leading individuals to interpret ordinary forgetfulness as a possible early sign of dementia (39, 52, 53, 61).

Psychological distress may further reinforce this process. Depression, anxiety, trait anxiety, and negative attitudes toward aging may increase vigilance toward cognitive changes and promote catastrophic interpretations (36). When individuals believe that dementia is not preventable or that coping resources are insufficient, fear may develop into avoidance behaviors, such as avoiding cognitive screening, delaying medical consultation, or avoiding dementia-related information (8, 35, 65). By contrast, good family communication, emotional support, and social connectedness may reduce uncertainty and buffer concerns about future disability, dependence, and lack of care (11, 23). Therefore, fear of dementia should be understood not only as an individual psychological issue, but also as a psychosocial phenomenon embedded in family and social support systems. However, important questions remain regarding the direction of these associations, the conditions under which fear motivates preventive behavior rather than avoidance, and whether these mechanisms differ across age groups, care settings, and cultural contexts. Longitudinal and comparative studies are needed to address these questions.

### 4.4 Cultural context and cross-cultural measurement issues

Cultural context may influence how people understand and express fear of dementia. Western studies more often emphasize personal autonomy, independent living, self-control, and continuity of identity; therefore, fear of dementia is frequently interpreted as a threat to independence and self-identity (36, 62). Instruments such as the FADS and ToDS are relatively consistent with this orientation because they include catastrophic attitudes, threats to self-identity, and existential threat (6, 41). By contrast, studies conducted primarily in China discussed fear of dementia in relation to family responsibility, intergenerational support, filial piety, caregiving burden, and stigma (21, 23, 59, 63). Supporting evidence from South Korea was limited, and the included Japanese studies did not directly support these themes. These observations should therefore not be generalized to East Asia as a whole.

However, these patterns should be interpreted cautiously. Most included studies were not designed as direct cross-cultural comparisons, and observed differences may reflect variations in research settings, sample characteristics, measurement content, and research questions rather than true cultural differences. Western-developed scales may also fail to fully capture culturally embedded concerns reported in Chinese populations, such as family burden, intergenerational responsibility, and stigma (21, 59, 63). Future studies should examine content equivalence, structural equivalence, and measurement invariance to avoid interpreting measurement or sample differences as cultural differences in fear of dementia (9, 41).

### 4.5 Public health and practical implications

The findings have implications for public health and practice. Given the predominantly observational nature of the available evidence, the following examples should be regarded as potential approaches for prospective evaluation rather than established practice recommendations. Future studies could evaluate whether fear of dementia may serve as a potential psychosocial factor in community-based cognitive health management, geriatric services, health examination centers, and health education settings. For individuals with subjective memory complaints, anxiety or depression, family history of dementia, living alone, or insufficient social support. Future research should examine whether assessing fear of dementia can help identify individuals who may benefit from dementia risk counseling, psychological support, or further cognitive assessment (35).

Potential approaches for evaluation may include health education that avoids relying solely on fear appeals, because overemphasizing the irreversible and severe consequences of dementia may increase anxiety, stigma, and avoidance of care. Approaches involving primary care providers, community health workers, and nurses could be evaluated to determine whether they help individuals distinguish normal forgetfulness from pathological cognitive decline, reduce catastrophic interpretations, and improve access to cognitive screening, management of modifiable risk factors, lifestyle interventions, and social support resources (50). Culturally adapted dementia education, anti-stigma initiatives, and community referral pathways could also be evaluated prospectively. In settings where concerns about becoming a family burden or experiencing stigma are prominent, family-participatory education, intergenerational communication, and linkage to community resources may warrant further evaluation.

### 4.6 Strengths and limitations

This review has several strengths. First, it included both Chinese- and English-language studies and covered multiple countries and regions, providing a relatively comprehensive synthesis of prevalence, measurement approaches, associated factors, and cultural contexts. Second, this review integrated not only measurement evidence, but also prevalence estimates, associated factors, and cultural interpretations. Third, the use of JBI appraisal and COSMIN-based evaluation enhanced the rigor of the methodological and measurement-property assessment (13, 14).

Several limitations should also be noted. First, most included studies were cross-sectional, limiting causal interpretation. Second, substantial heterogeneity in populations, instruments, scoring methods, cut-off criteria, and outcome reporting precluded meta-analysis. Third, some studies did not adequately report sample selection, sample size estimation, non-response, confounding control, or statistical details, which may have affected the strength of evidence. Although most JBI-appraised studies received favorable ratings, the overall confidence in the evidence was limited by the predominantly cross-sectional designs, methodological and measurement heterogeneity, incomplete evidence across several COSMIN domains, and the small or indirect evidence bases supporting some conclusions. Confidence was relatively greater in the direction of the repeatedly observed associations involving anxiety/depression and subjective cognitive concerns than in their magnitude or causal interpretation; by contrast, confidence in prevalence estimates, social-support findings, and broader cultural patterns remained limited. In addition, although the inclusion of theses and dissertations broadened the evidence base, differences in peer-review and reporting standards may reduce confidence in some findings; these sources were therefore assessed using the same JBI criteria as journal articles and interpreted cautiously. Finally, this review included only Chinese- and English-language literature, which may have introduced language bias. The cultural interpretation was based predominantly on Chinese studies, with limited supporting evidence from South Korea and no direct supporting evidence from the included Japanese studies; these findings should therefore not be generalized to East Asia as a whole and require validation through high-quality comparative research.

## 5 Conclusion

Fear of dementia and related constructs were frequently reported across diverse adult populations without diagnosed dementia; however, the available evidence does not permit a reliable estimate of their prevalence or magnitude. Reported levels were associated with demographic characteristics, subjective cognitive perceptions, psychological status, prior exposure to dementia, social support, and cultural context, although the consistency and strength of these associations varied. Although existing measurement instruments have established a certain foundation of reliability and validity, further improvement is still needed in terms of content validity, cross-cultural measurement invariance, measurement error, and responsiveness. Future research should prioritize longitudinal studies, cross-cultural validation of measurement instruments, and prospective evaluation of interventions and community-based assessment approaches aimed at reducing excessive fear of dementia and improving engagement, timely help-seeking, and psychological outcomes.

## Data Availability

The original contributions presented in the study are included in the article/Supplementary material, further inquiries can be directed to the corresponding author.
