# Supplementary material for: Fear of dementia in adults without diagnosed dementia: an updated systematic review of prevalence, measurement, and correlates
Source: Front Public Health. 2026 Aug 28;14:1930480. doi: 10.3389/fpubh.2026.1930480 (PMC13561815; doi:10.3389/fpubh.2026.1930480)
Supplement: Supplementary file 1 [file Table_1.DOCX]

**Supplementary Table 1. Full search strategy**

Note. All database searches were conducted from inception to April 10, 2026.

**Search period:** from database inception to April 10, 2026. Eligibility was restricted to quantitative original studies published in Chinese or English that focused on dementia fear, dementia worry, dementia-related anxiety, concern about developing dementia, perceived threat of Alzheimer's disease, fear of memory loss, or related constructs. The final electronic search included eight databases. Record counts below correspond to the final PRISMA total.

**A. Summary of electronic search results**

| **Database** | **Search field / approach** | **Records identified (n)** |
| --- | --- | --- |
| PubMed | Title/Abstract proximity search | 2,108 |
| Web of Science | Topic search with NEAR/4 | 4,151 |
| PsycINFO | Title/Abstract search with N4 | 354 |
| Cochrane Library | Title/Abstract/Keyword search with NEAR/4 | 283 |
| WanFang Data | Title or keyword / title search | 179 |
| VIP | Title/keyword search | 171 |
| CBM | Smart title search | 147 |
| CNKI | Title/keyword phrase search | 74 |
| **Total records identified through database searching** | **Total records identified through database searching** | **7,467** |

**B. Detailed search strategy by database**

**1. PubMed**

| **Search field / approach** | Title/Abstract proximity search |
| --- | --- |
| **Search strategy** | (  "dementia worry"[tiab:~4] OR "dementia worries"[tiab:~4] OR "dementia fear"[tiab:~4] OR  "dementia fears"[tiab:~4] OR "dementia anxiety"[tiab:~4] OR "dementia concern"[tiab:~4] OR  "dementia concerns"[tiab:~4] OR "dementia threat"[tiab:~4] OR  "alzheimer worry"[tiab:~4] OR "alzheimer worries"[tiab:~4] OR "alzheimer fear"[tiab:~4] OR  "alzheimer fears"[tiab:~4] OR "alzheimer anxiety"[tiab:~4] OR "alzheimer concern"[tiab:~4] OR  "alzheimer concerns"[tiab:~4] OR "alzheimer threat"[tiab:~4] OR  "anticipatory dementia"[tiab] ) |
| **Records identified** | 2,108 |

**2. Web of Science**

| **Search field / approach** | Topic search |
| --- | --- |
| **Search strategy** | (dementia NEAR/4 worr*) OR (dementia NEAR/4 fear*) OR (dementia NEAR/4 anxi*) OR (dementia NEAR/4 concern*) OR (dementia NEAR/4 threat*) OR (alzheimer* NEAR/4 worr*) OR (alzheimer* NEAR/4 fear*) OR (alzheimer* NEAR/4 anxi*) OR (alzheimer* NEAR/4 concern*) OR (alzheimer* NEAR/4 threat*) OR ("anticipatory dementia") |
| **Records identified** | 4,151 |

**3. PsycINFO**

| **Search field / approach** | Title/Abstract search |
| --- | --- |
| **Search strategy** | (dementia N4 worr*) OR (dementia N4 fear*) OR (dementia N4 anxi*) OR (dementia N4 concern*) OR (dementia N4 threat*) OR (alzheimer* N4 worr*) OR (alzheimer* N4 fear*) OR (alzheimer* N4 anxi*) OR (alzheimer* N4 concern*) OR (alzheimer* N4 threat*) OR "anticipatory dementia" |
| **Records identified** | 354 |

**4. Cochrane Library**

| **Search field / approach** | Title/Abstract/Keyword search |
| --- | --- |
| **Search strategy** | (dementia NEAR/4 worr*) OR (dementia NEAR/4 fear*) OR (dementia NEAR/4 anxi*) OR (dementia NEAR/4 concern*) OR (dementia NEAR/4 threat*) OR (alzheimer* NEAR/4 worr*) OR (alzheimer* NEAR/4 fear*) OR (alzheimer* NEAR/4 anxi*) OR (alzheimer* NEAR/4 concern*) OR (alzheimer* NEAR/4 threat*) OR "anticipatory dementia" |
| **Records identified** | 283 |

**5. WanFang Data**

| **Search field / approach** | Title/keyword and title search |
| --- | --- |
| **Search strategy** | 题名或关键词：(痴呆 OR 阿尔茨海默 OR 老年痴呆) AND 题名：(恐惧 OR 担忧 OR 忧虑 OR 焦虑 OR 害怕 OR 恐慌) |
| **Records identified** | 179 |

**6. VIP**

| **Search field / approach** | Title/keyword search |
| --- | --- |
| **Search strategy** | (痴呆 OR 阿尔茨海默 OR 老年痴呆) AND (恐惧 OR 担忧 OR 忧虑 OR 焦虑 OR 害怕) |
| **Records identified** | 171 |

**7. CBM**

| **Search field / approach** | Smart title search |
| --- | --- |
| **Search strategy** | ("痴呆"[标题:智能] OR "阿尔茨海默"[标题:智能] OR "老年痴呆"[标题:智能]) AND ("恐惧"[标题:智能] OR "担忧"[标题:智能] OR "忧虑"[标题:智能] OR "焦虑"[标题:智能] OR "害怕"[标题:智能] OR "恐慌"[标题:智能]) |
| **Records identified** | 147 |

**8. CNKI**

| **Search field / approach** | Title/keyword phrase search |
| --- | --- |
| **Search strategy** | "痴呆恐惧" OR "老年痴呆恐惧" OR "阿尔茨海默病恐惧" OR "痴呆担忧" OR "担心患痴呆" OR "对痴呆的焦虑" OR "前瞻性痴呆恐惧" |
| **Records identified** | 74 |

**Notes.** NEAR/4 and N4 indicate that the search terms were required to occur within four words of each other. Truncation (*) was used where supported by the database. Chinese database strategies were retained in Chinese to preserve the exact search wording. CINAHL and Embase trial searches recorded in the working file were not part of the final eight-database PRISMA count and are therefore not included in this supplementary table.
