# Supplementary material for: Fear of dementia in adults without diagnosed dementia: an updated systematic review of prevalence, measurement, and correlates
Source: Front Public Health. 2026 Aug 28;14:1930480. doi: 10.3389/fpubh.2026.1930480 (PMC13561815; doi:10.3389/fpubh.2026.1930480)
Supplement: Supplementary file 2 [file Table_2.DOCX]

**Supplementary Table 2. Characteristics of included studies (n = 56).**

| **No.** | **Ref.** | **Study** | **Country/region** | **Core term** | **Design** | **Setting/participants** | **Sample size and age** | **Measure** | **Baseline result** | **Psychometric properties** |
| --- | --- | --- | --- | --- | --- | --- | --- | --- | --- | --- |
| 1 | (57) | Arai et al., 2012 | Japan | Concern about developing dementia | Cross-sectional study | General public | N = 2,500; age >=20 years | Single-item self-report question | 62.1% of participants reported concern | NA |
| 2 | (64) | Ashworth et al., 2022 | United Kingdom | Worry about developing dementia | Cross-sectional study | Community-dwelling adults | N = 311; mean age = 46.89 years | NRS | Mean score: 3.92 ± 3.09 | NA |
| 3 | (29) | Bai et al., 2024 | China | Fear of dementia | Cross-sectional study | Hospitalized older adults | N = 458; 60-91 years; mean age = 69.22 ± 6.89 years | FODS (Chinese version) | Median score: 54.00 | NA |
| 4 | (37) | Bowen et al., 2019 | Germany | Dementia worry | Cross-sectional study | Community-dwelling middle-aged and older adults | N = 219; mean age = 65.5 years | DWS (10 items) | Prevalence: 41.1% | NA |
| 5 | (41) | Cheston et al., 2022 | United Kingdom | Threat of dementia | Scale validation study | Adults and university students | N = 248 and N = 99 | ToDS | University student sample: 68.30 ± 12.14 | One-factor structure; explained variance = 49.47%; Cronbach's alpha = 0.91 |
| 6 | (34) | Cutler and Hodgson, 2001 | United States | Concern about developing Alzheimer's disease | Cross-sectional study | Middle-aged adults | N = 258; mean age = 49.7 years | Single-item self-report question | 24.0% reported being “very” concerned | NA |
| 7 | (65) | Dai et al., 2023 | China | Fear of dementia | Cross-sectional study | Community-dwelling older adults | N = 259; age >=60 years | FODS (Chinese version) | Mean score: 55.32 ± 9.42 | NA |
| 8 | (18) | Deng et al., 2025 | China | Fear of memory loss | Scale validation study | Community-dwelling older adults | N = 310; age >=60 years | FAM (Chinese version) | NR | Two-factor structure (fear/avoidance); S-CVI = 0.96; Cronbach's alpha = 0.934 |
| 9 | (43) | Ebert et al., 2020 | United States | Personal dementia fear | Cross-sectional study | Community-dwelling adults | N = 616; mean age = 46.03 years | FADS (partial extraction) | Personal dementia fear: 2.36 ± 0.78 | NA |
| 10 | (56) | Farina et al., 2022 | International/multinational | Fear of memory loss | Scale validation study | General older adults | NR | FAM (preliminary version) | NR | Three-factor structure; total-scale Cronbach's alpha = 0.82; good concurrent validity |
| 11 | (8) | Farina et al., 2023 | International/multinational | Fear of memory loss | Scale validation study | Community-dwelling older adults | N = 813; age >55 years | FAM | Mean score: 27.2 in the US sample and 24.6 in the international sample | Two-factor structure (fear/avoidance); Cronbach's alpha >=0.80; good concurrent validity |
| 12 | (58) | French, 2008 | United States | Fear of developing Alzheimer's disease | Cross-sectional study | Community-dwelling older adults | N = 50; 65-84 years | FADS | Mean score: 25.44 ± 20.91 | NA |
| 13 | (6) | French et al., 2012 | United States | Anticipatory dementia/fear of Alzheimer's disease | Scale validation study | Community-dwelling older adults | N = 101; 65-91 years | FADS | NR | Three-factor model (general fear, physical symptoms, catastrophic attitude); Cronbach's alpha = 0.94; good validity |
| 14 | (27) | Gu, 2024 | China | Fear of dementia | Cross-sectional study | Older adults in long-term care institutions | N = 140; age >=60 years; mean age = 78.23 years | FODS (18-item version) | Mean score ranged from 64.71 to 67.12 | NA |
| 15 | (19) | Guo et al., 2025 | China | Fear of dementia | Cross-sectional study | Hospitalized older adults | N = 263; age >=60 years; mean age = 66.73 ± 5.46 years | FODS (18-item version) | Median total score: 76.00 | NA |
| 16 | (39) | Hajek and König, 2020a | Germany | Perceived own risk | Cross-sectional study | Community-dwelling adults from the general population | N = 1,542; mean age = 51.1 years | Single-item self-rating scale | Mean perceived risk score: 2.4 ± 0.9 | NA |
| 17 | (55) | Hajek and König, 2020b | Germany | Fear of dementia | Cross-sectional study | General adult population | N = 1,498; mean age = 50.9 years | Single-item self-report question | 15.7% reported severe fear | NA |
| 18 | (26) | Han et al., 2023 | China | Fear of dementia | Cross-sectional study | Older adults in long-term care institutions | N = 550; 60-94 years; mean age = 79.57 ± 7.47 years | FODS (18-item version) | Mean score: 56.50 ± 10.73; 68.0% at moderate-to-high levels | NA |
| 19 | (49) | Joo et al., 2021 | South Korea | Dementia worry | Cross-sectional study | Community-dwelling older adults | N = 1,869; age >=60 years | Self-report questionnaire | NR | NA |
| 20 | (35) | Khalil et al., 2025 | Middle East | Fear of dementia | Cross-sectional study | Community-dwelling older adults | N = 1,067 | Dementia Literacy Questionnaire; Personal Dementia Fear; MCLHB-DRR; PRISM-PC | Dementia literacy total score: 2.88 ± 1.82; personal dementia fear: 27.16 ± 8.91 | NA |
| 21 | (52) | Ran and Chang, 2020 | South Korea | Fear of dementia | Cross-sectional study | Community-dwelling middle-aged and older adults | N = 156; age >=40 years | Structured questionnaire | Mean score: 1.49 ± 0.87 (converted to a 4-point scale) | NA |
| 22 | (61) | Laforce and McLean, 2005 | Canada | Fear of developing Alzheimer's disease | Cross-sectional study | Young and older adults | N = 245 | Custom single-item questionnaire | Fear was significantly higher in younger participants than in older participants | NA |
| 23 | (36) | Larry et al., 2025 | United States | Dementia worry | Cross-sectional study | Young adults | N = 140; 18-25 years | DWS (modified version) | Mean score: 18.33 ± 7.32 | NA |
| 24 | (45) | Lathrop, 2017 | United States | Dementia worry | Cross-sectional study | Community-dwelling middle-aged and older adults | N = 122 | 1-5 rating scale | Predicted model score was approximately 3 | NA |
| 25 | (40) | Lee, 2020 | South Korea | Fear of dementia | Scale validation study | Community-dwelling older adults | N = 233 | FODS | NR | Three dimensions and five factors; explained variance = 78.44%; good model fit |
| 26 | (44) | Lee et al., 2021 | United States | Dementia worry | Cross-sectional study | Community-dwelling middle-aged and older adults | N = 126; mean age = 65.11 years | DWS | Genetic exposure group: 34.60 ± 12.16; non-genetic exposure group: 27.67 ± 6.94; no exposure group: 29.85 ± 11.08 | NA |
| 27 | (24) | Li, 2024 | China | Fear of dementia | Cross-sectional study | Community-dwelling older adults | N = 339; age >=60 years; mean age = 67.0 ± 6.5 years | FODS (18-item version) | Mean score: 58.18 ± 12.46 | NA |
| 28 | (51) | Lim et al., 2021 | South Korea | Anxiety about dementia | Cross-sectional study | Community-dwelling older adults | N = 182; age >=65 years | VAS | Mean score: 4.16 ± 3.61 | NA |
| 29 | (30) | Lin et al., 2025a | Hong Kong, China | Fear of Alzheimer's disease | Scale validation study | Community-dwelling middle-aged and older adults | N = 196; mean age = 67.82 years | FADS (Chinese version) | Mean score: 55.96 ± 22.93 | Supported the three-factor structure; Cronbach's alpha = 0.912; test-retest ICC = 0.793 |
| 30 | (7) | Lin et al., 2025b | Hong Kong, China | Dementia-related fear | Cross-sectional study | Community-dwelling middle-aged and older adults | N = 200; mean age = 67.58 years | FADS (Chinese version) | Mean score: 57.52 ± 23.61 | NA |
| 31 | (59) | Liu et al., 2022 | China | Perceived dementia worry | Cross-sectional study | Rural adults | N = 577 | Self-developed dementia worry scale (9 items); self-developed dementia beliefs scale (10 items); ADKS (16 items) | NR | NA |
| 32 | (31) | Maxfield and Greenberg, 2021 | United States | Dementia-related anxiety | Cross-sectional study | Community-dwelling middle-aged and older adults | N = 183; mean age = 59.57 years | DWS | Mean score: 3.39 ± 0.69 (5-point scale) | NA |
| 33 | (54) | Meyer et al., 2024 | Germany | Fear of dementia | Cross-sectional study | General adult population | N = 3,890; 40-70 years; mean age = 54.9 years | Single-item question | 12.7% reported severe fear | NA |
| 34 | (66) | Moon et al., 2014 | South Korea | Anticipatory dementia | Scale validation study | Healthy older volunteers | N = 108; mean age = 68.2 years | K-FADS | Mean score: 35.3 ± 25.2 in women and 33.4 ± 26.2 in men | Confirmed three-factor structure; Cronbach's alpha = 0.96; test-retest ICC = 0.98 |
| 35 | (50) | Nakayama et al., 2025 | Japan | Dementia worry | Cross-sectional study | Community-dwelling middle-aged and older adults | N = 299; mean age = 64.8 years | DWAS | Mean score: 22.7 ± 13.7 | NA |
| 36 | (60) | Ostergren et al., 2017 | United States | Perceived threat of Alzheimer's disease | Cross-sectional study | Community-dwelling adults | N = 1,641; mean age = 64.4 years | Perceived Alzheimer's disease threat measure | Perceived threat was significantly higher in participants aged 50-74 years than in those aged >=75 years | NA |
| 37 | (42) | Page et al., 2019 | United States | Fear of dementia | Scale validation study | Cognitively intact middle-aged and older adults | Baseline N = 734; follow-up N = 226 | FOD | NR | Three-dimensional structure; good construct validity |
| 38 | (53) | Rahman, 2016 | United States | Fear of Alzheimer's disease | Scale validation study | Community-dwelling middle-aged and older adults | N = 93 | FADS; ADC | NR | Established construct validity and principal component results for the ADC |
| 39 | (25) | Ran, 2023 | China | Fear of dementia | Cross-sectional study | Community-dwelling older adults | N = 761; 60-92 years; mean age = 72.89 ± 7.60 years | FODS (18-item version) | Mean score: 52.59 ± 15.10 | NA |
| 40 | (15) | Roberts and Maxfield, 2021 | United States | Dementia worry | Scale validation study | General adults | Total N = 592 (Study 1: N = 197; Study 2: N = 395) | DWS; MDWS | NR | One-factor structure; Cronbach's alpha >=0.95; good test-retest reliability (r = 0.90) |
| 41 | (11) | Ryu and Park, 2019 | South Korea | Dementia worry | Cross-sectional study | Community-dwelling middle-aged and older adults | N = 205 | NR | Dementia worry was significantly higher in older adults than in middle-aged adults | NA |
| 42 | (47) | Said, 2018 | United Kingdom | Fear of dementia | Cross-sectional study | Community adults undergoing screening | N = 338; 50-65 years | FADS (partial extraction) | Participants with family history: 35.43; those without family history: 32.25 | NA |
| 43 | (17) | Tan et al., 2025a | China | Fear of dementia | Cross-sectional study | Rural older adults | N = 349; 60-95 years; mean age = 71.70 ± 7.19 years | FADS (30-item version) | Mean score: 36.48 ± 24.94 | NA |
| 44 | (21) | Tan et al., 2025b | China | Fear of dementia | Cross-sectional study | Rural older adults | N = 349; 60-95 years; mean age = 71.70 ± 7.19 years | FADS (30-item version) | Mean score: 36.48 ± 24.94 | NA |
| 45 | (20) | Tan et al., 2026 | China | Fear of dementia | Cross-sectional study | Rural older adults | N = 349; 60-95 years; mean age = 71.70 ± 7.19 years | FADS (30-item version) | Mean score: 36.48 ± 24.94; moderate group accounted for 67.33% | NA |
| 46 | (22) | Wang et al., 2023 | China | Fear of dementia | Cross-sectional study | Community-dwelling older adults | N = 384; age >=60 years | FODS (18-item version) | Mean score: 57.96 ± 12.74 | NA |
| 47 | (32) | Werner, 2002 | Israel | Concern about developing Alzheimer's disease | Cross-sectional study | Community-dwelling adults | N = 186; mean age = 64.1 years | Self-developed single-item question | 46% of participants were very concerned | NA |
| 48 | (62) | Werner et al., 2026 | Israel | Dementia-related anxiety | Cross-sectional study | Jewish adults | N = 280; mean age = 41.6 years | MDWS | Mean score: 19.9 ± 9.0; two major subgroups identified | NA |
| 49 | (48) | Whitty, 2020 | United Kingdom | Fear of dementia | Cross-sectional study | Healthy community-dwelling adults | Baseline N = 1,134; 50-65 years | Brief-FoD | Mean score: 21.03 ± 11.75 | NA |
| 50 | (23) | Wu et al., 2022 | China | Fear of dementia | Cross-sectional study | Community-dwelling older adults | N = 621; 60-84 years; mean age = 69.98 ± 6.09 years | FODS (Chinese version) | Mean score: 55.65 ± 13.56 | NA |
| 51 | (63) | Wu et al., 2025 | China | Fear of dementia | Cross-sectional study | Community-dwelling older adults | N = 656; mean age = 70.05 years | FODS | Mean score: 55.05 ± 12.81 | NA |
| 52 | (16) | Xu et al., 2021 | China | Fear of dementia | Scale validation study | Community-dwelling older adults | N = 230; 60-89 years; mean age = 68.53 ± 6.80 years | FODS | NR | Four common factors extracted; S-CVI = 0.934; Cronbach's alpha = 0.893 |
| 53 | (38) | Yun, 2019 | United States | Dementia worry | Cross-sectional study | Middle-aged and older adults | N = 122; mean age = 65.66 years | DWS | Mean score: 3.34 in men and 3.44 in women | NA |
| 54 | (46) | Yun and Choi, 2025 | South Korea | Fear of dementia | Cross-sectional study | Community-dwelling older adults | N = 199; age >=65 years | K-FADS | Mean score: 85.02 ± 23.79 | NA |
| 55 | (33) | Yun and Maxfield, 2020 | United States | Dementia-related anxiety | Cross-sectional study | General adults | N = 647 (Study 1: N = 295; Study 2: N = 352) | DRA scale | Study 2 sample: DRA mean score = 1.93 ± 0.86 | NA |
| 56 | (28) | Zhang et al., 2022 | China | Fear of Alzheimer's disease | Scale validation study | Community-dwelling older adults | N = 186; 60-86 years; mean age = 67.84 ± 4.03 years | FADS (Chinese version) | NR | Three common factors extracted; S-CVI = 0.94; Cronbach's alpha = 0.934 |

**Note.** Reference numbers correspond to the reference list of the main manuscript. NA = not applicable; NR = not reported or unclear in the available information. FODS = Fear of Dementia Scale; FADS = Fear of Alzheimer's Disease Scale; K-FADS = Korean version of FADS; DWS = Dementia Worry Scale; MDWS = Modified Dementia Worry Scale; DWAS = Dementia Worry Assessment Scale; FAM = Fear and Avoidance of Memory Loss scale; ToDS = Threat of Dementia Scale; VAS = Visual Analogue Scale; NRS = Numerical Rating Scale; ADC = Anticipatory Dementia Checklist; DRA = Dementia-Related Anxiety Scale.

**Terminology note.** Terms such as dementia fear, dementia worry, dementia-related anxiety, and threat of dementia are used in the included studies to describe negative emotional responses to the possibility of developing dementia or related cognitive decline. The wording follows the original terminology used in each study.
