# Supplementary material for: Fear of dementia in adults without diagnosed dementia: an updated systematic review of prevalence, measurement, and correlates
Source: Front Public Health. 2026 Aug 28;14:1930480. doi: 10.3389/fpubh.2026.1930480 (PMC13561815; doi:10.3389/fpubh.2026.1930480)
Supplement: Supplementary file 3 [file Table_3.DOCX]

**Supplementary Table 3. JBI methodological quality appraisal of cross-sectional studies**

This table reports the study-level methodological quality appraisal using the JBI Critical Appraisal Checklist for Analytical Cross-Sectional Studies. A total of 43 analytical cross-sectional studies were appraised using the JBI checklist. Instrument-development/validation studies and other designs were not included in this JBI table. Quality ratings were defined as high (≥6 Yes responses), moderate (4–5 Yes responses), and low (≤3 Yes responses).

**Checklist items and response codes**

| **Item** | **JBI checklist question** |
| --- | --- |
| Q1 | Were the criteria for inclusion in the sample clearly defined? |
| Q2 | Were the study subjects and the setting described in detail? |
| Q3 | Was the exposure measured in a valid and reliable way? |
| Q4 | Were objective, standard criteria used for measurement of the condition? |
| Q5 | Were confounding factors identified? |
| Q6 | Were strategies to deal with confounding factors stated? |
| Q7 | Were the outcomes measured in a valid and reliable way? |
| Q8 | Was appropriate statistical analysis used? |

Y = Yes; N = No; U = Unclear; NA = Not applicable.

**Summary of quality ratings**

| **Quality rating** | **Number of studies** | **Percentage** |
| --- | --- | --- |
| High | 35 | 81.4% |
| Moderate | 5 | 11.6% |
| Low | 3 | 7.0% |
| **Total** | **43** | **100.0%** |

**Response distribution by JBI item**

| **Item** | **Y** | **N** | **U** | **NA** |
| --- | --- | --- | --- | --- |
| Q1 | 42 | 0 | 1 | 0 |
| Q2 | 42 | 0 | 1 | 0 |
| Q3 | 34 | 0 | 9 | 0 |
| Q4 | 34 | 1 | 8 | 0 |
| Q5 | 39 | 2 | 1 | 1 |
| Q6 | 39 | 2 | 1 | 1 |
| Q7 | 34 | 0 | 9 | 0 |
| Q8 | 43 | 0 | 0 | 0 |

**Study-level JBI appraisal results**

| **Ref.** | **Study** | **Q1** | **Q2** | **Q3** | **Q4** | **Q5** | **Q6** | **Q7** | **Q8** | **Yes score** | **Quality rating** |
| --- | --- | --- | --- | --- | --- | --- | --- | --- | --- | --- | --- |
| (57) | Arai et al., 2012 | Y | Y | U | U | Y | Y | U | Y | 5 | Moderate |
| (64) | Ashworth et al., 2022 | Y | Y | U | Y | N | N | Y | Y | 5 | Moderate |
| (29) | Bai et al., 2024 | Y | Y | Y | Y | Y | Y | Y | Y | 8 | High |
| (37) | Bowen et al., 2019 | Y | Y | Y | Y | Y | Y | Y | Y | 8 | High |
| (34) | Cutler and Hodgson, 2001 | Y | Y | U | U | Y | Y | U | Y | 5 | Moderate |
| (65) | Dai et al., 2023 | Y | Y | Y | Y | Y | Y | Y | Y | 8 | High |
| (43) | Ebert et al., 2020 | Y | Y | Y | Y | Y | Y | Y | Y | 8 | High |
| (58) | French, 2008 | Y | Y | Y | Y | Y | Y | Y | Y | 8 | High |
| (19) | Guo et al., 2025 | Y | Y | Y | Y | Y | Y | Y | Y | 8 | High |
| (55) | Hajek and König, 2020a | Y | Y | U | U | Y | Y | U | Y | 5 | Moderate |
| (39) | Hajek and König, 2020b | Y | Y | Y | U | Y | Y | U | Y | 6 | High |
| (26) | Han et al., 2023 | Y | Y | Y | Y | Y | Y | Y | Y | 8 | High |
| (49) | Joo et al., 2021 | Y | Y | U | N | Y | Y | U | Y | 5 | Moderate |
| (35) | Khalil et al., 2025 | Y | Y | Y | Y | Y | Y | Y | Y | 8 | High |
| (52) | Ran and Chang, 2020 | Y | Y | Y | Y | Y | Y | Y | Y | 8 | High |
| (61) | Laforce and McLean, 2005 | Y | Y | U | U | U | U | U | Y | 3 | Low |
| (36) | Larry et al., 2025 | Y | Y | Y | Y | Y | Y | Y | Y | 8 | High |
| (45) | Lathrop, 2017 | U | U | U | U | Y | Y | U | Y | 3 | Low |
| (44) | Lee et al., 2020 | Y | Y | Y | Y | Y | Y | Y | Y | 8 | High |
| (24) | Li, 2024 | Y | Y | Y | Y | Y | Y | Y | Y | 8 | High |
| (51) | Lim et al., 2021 | Y | Y | U | Y | Y | Y | Y | Y | 7 | High |
| (7) | Lin et al., 2025 | Y | Y | Y | Y | Y | Y | Y | Y | 8 | High |
| (59) | Liu et al., 2022 | Y | Y | Y | Y | Y | Y | Y | Y | 8 | High |
| (31) | Maxfield and Greenberg, 2021 | Y | Y | Y | Y | Y | Y | Y | Y | 8 | High |
| (54) | Meyer et al., 2024 | Y | Y | Y | U | Y | Y | U | Y | 6 | High |
| (50) | Nakayama et al., 2025 | Y | Y | Y | Y | Y | Y | Y | Y | 8 | High |
| (60) | Ostergren et al., 2017 | Y | Y | Y | Y | Y | Y | Y | Y | 8 | High |
| (25) | Ran, 2023 | Y | Y | Y | Y | Y | Y | Y | Y | 8 | High |
| (11) | Ryu and Park, 2019 | Y | Y | Y | Y | Y | Y | Y | Y | 8 | High |
| (47) | Said, 2018 | Y | Y | Y | Y | Y | Y | Y | Y | 8 | High |
| (21) | Tan et al., 2025a | Y | Y | Y | Y | Y | Y | Y | Y | 8 | High |
| (17) | Tan et al., 2025b | Y | Y | Y | Y | Y | Y | Y | Y | 8 | High |
| (20) | Tan et al., 2026 | Y | Y | Y | Y | Y | Y | Y | Y | 8 | High |
| (22) | Wang et al., 2023 | Y | Y | Y | Y | Y | Y | Y | Y | 8 | High |
| (32) | Werner, 2002 | Y | Y | U | U | N | N | U | Y | 3 | Low |
| (62) | Werner et al., 2026 | Y | Y | Y | Y | NA | NA | Y | Y | 6 (NA not counted) | High |
| (48) | Whitty, 2020 | Y | Y | Y | Y | Y | Y | Y | Y | 8 | High |
| (23) | Wu et al., 2022 | Y | Y | Y | Y | Y | Y | Y | Y | 8 | High |
| (63) | Wu et al., 2025 | Y | Y | Y | Y | Y | Y | Y | Y | 8 | High |
| (38) | Yun, 2019 | Y | Y | Y | Y | Y | Y | Y | Y | 8 | High |
| (33) | Yun and Maxfield, 2020 (Study 1) | Y | Y | Y | Y | Y | Y | Y | Y | 8 | High |
| (33) | Yun and Maxfield, 2020 (Study 2) | Y | Y | Y | Y | Y | Y | Y | Y | 8 | High |
| (46) | Yun and Choi, 2025 | Y | Y | Y | Y | Y | Y | Y | Y | 8 | High |

Note. Ref. = reference number in the main reference list. The Yes score indicates the number of “Yes” responses across applicable JBI items. NA responses were not counted in the denominator when calculating the Yes score. For Werner et al. (2026), Q5 and Q6 were judged not applicable. Instrument-development/validation studies were not appraised using the cross-sectional JBI checklist.
