# Supplementary material for: Fear of dementia in adults without diagnosed dementia: an updated systematic review of prevalence, measurement, and correlates
Source: Front Public Health. 2026 Aug 28;14:1930480. doi: 10.3389/fpubh.2026.1930480 (PMC13561815; doi:10.3389/fpubh.2026.1930480)
Supplement: Supplementary file 4 [file Table_4.DOCX]

Supplementary Table 4. COSMIN-based evaluation of psychometric evidence for dementia fear and fear-of-memory-loss instruments

This supplementary table summarizes COSMIN-based evidence extracted from instrument-development, translation, or validation studies. It indicates whether each measurement property was evaluated and provides a concise description of the main available psychometric evidence.

Summary of available evidence across COSMIN domains

| **COSMIN domain** | **Main appraisal finding** |
| --- | --- |
| **PROM development/translation** | Reported for all instruments; original instruments described item generation, while translated versions described forward/back translation and cultural adaptation. |
| **Content validity** | Quantitative CVI was reported in several Chinese versions; many original studies relied on theory, expert review, brainstorming or pilot testing without full quantitative CVI reporting. |
| **Structural validity** | Evaluated in all included instrument-development or validation studies, mainly using EFA, PCA or CFA. |
| **Internal consistency** | Reported in all studies; most total-scale Cronbach alpha values were acceptable to excellent. |
| **Cross-cultural validity/measurement invariance** | Translation and cultural adaptation were reported for Chinese and Korean versions, but formal measurement invariance testing was generally absent. |
| **Reliability** | Test-retest reliability was reported for selected instruments or translated versions, including MDWS, Chinese FADS, ToDS, K-FADS, Chinese FODS and Chinese FAM. |
| **Measurement error** | Not assessed in the extracted evidence; SEM, SDC or MIC were generally not reported. |
| **Criterion validity** | Generally not assessed because no accepted gold standard exists; some correlations with related scales were better interpreted as convergent validity. |
| **Hypothesis testing/construct validity** | Frequently supported by expected associations with anxiety, depression, memory complaints, perceived susceptibility/severity, FADS/FODS and other theoretically related variables. |
| **Responsiveness** | Not assessed; available studies were mainly cross-sectional or did not evaluate sensitivity to clinical/intervention change. |

Detailed COSMIN-domain evidence by instrument

| **Ref.** | **Study/instrument** | **PROM development/translation** | **Content validity** | **Structural validity** | **Internal consistency** | **Cross-cultural validity/measurement invariance** | **Reliability** | **Measurement error** | **Criterion validity** | **Hypothesis testing/construct validity** | **Responsiveness** |
| --- | --- | --- | --- | --- | --- | --- | --- | --- | --- | --- | --- |
| (30) | Lin et al. (2025) - Chinese FADS | Translated and culturally adapted from the original FADS; five older adults completed cognitive interviews and wording revisions. | 4 experts; S-CVI=0.833; I-CVI=0.80-1.00. | EFA supported the original 3-factor structure; variance explained=75.35%. | Total Cronbach alpha=0.912; subscale alpha=0.929-0.969. | Brislin translation/back-translation; cultural adaptation such as replacing Western celebrity examples with Gao Kun. Formal invariance not reported. | 2-week ICC=0.793 (n=32). | Not assessed. | Not assessed because no gold standard was available. | Supported by associations with perceived susceptibility/severity and group differences by dementia contact history. | Not assessed. |
| (15) | Roberts and Maxfield (2021) - Modified DWS/MDWS | Adapted terminology from dementia to ADRD to improve construct precision. | No quantitative CVI; changes were theory-driven. | Single-factor structure supported in two studies; variance explained about 66%. | Cronbach alpha=0.95-0.96. | Not assessed; mainly US MTurk adult samples. | 4-week r=0.90; 8-week r=0.78. | Not assessed. | Not assessed. | Supported by significant correlations with fear of dementia, subjective memory decline, anxiety, health anxiety and neuroticism. | Not assessed. |
| (42) | Page et al. (2019) - FOD scale | Generated from clinical experience, brainstorming and prior qualitative literature; initial 39-item pool. | No quantitative CVI; face and content evidence was limited. | PCA suggested 3 factors: burden/loss, quality of life, perceived social/cognitive loss; variance explained=25.3%. | Total Cronbach alpha=0.98; subscale alpha=0.86-0.97. | Not assessed; sample predominantly White and no formal cross-cultural validation. | Longitudinal data existed, but factor structure changed and test-retest reliability was not calculated. | Not assessed. | Not assessed. | Supported by correlations with aging anxiety and neuroticism, and negative correlation with conscientiousness. | Not assessed. |
| (40) | Lee and Jung (2020) - FODS | Developed using fear expectation theory, literature review and concept analysis; initial 53-item pool. | Two rounds of expert assessment by 12 experts, but specific CVI values were not reported. | EFA identified 5 factors across 3 dimensions; cumulative variance=78.44%. | Total Cronbach alpha=0.927; subscale alpha=0.849-0.923; Rasch separation indices were favorable. | Developed for Korean older adults; no cross-cultural measurement invariance testing. | Not reported. | Not reported; Rasch separation indices were provided but absolute measurement error was not. | Correlations with K-FADS and NRS were reported; best interpreted as convergent validity rather than criterion validity. | Known-groups validity supported by significant differences between participants who reported fear of dementia and those who did not. | Not assessed. |
| (53) | Rahman (2016) - ADI | Developed from the Health Belief Model; initial 50-item instrument. | No quantitative CVI; based mainly on theoretical framework. | PCA with parallel analysis extracted 6 components; cumulative variance=52.48%. | Total Cronbach alpha=0.896. | Not assessed. | Not reported. | Not assessed. | Not assessed. | Supported by correlations with FADS, subjective memory complaints, trait anxiety and depression. | Not assessed. |
| (56) | Farina et al. (2022) - FAM preliminary version | Developed using existing fear scales, professional/patient interviews and pilot testing with older adults; 23 items. | No quantitative CVI; content was supported by interviews and pilot testing. | EFA identified 3 factors: fear-avoidance, problematic beliefs and emotional resilience; variance explained >50%. | Total Cronbach alpha=0.82; factor 1 alpha=0.87; other factors had lower alpha. | Not assessed; sample largely White/Irish English-speaking adults. | Not reported. | Not assessed. | Not assessed. | Supported by correlations with GAI, memory failures and quality of life. | Not assessed. |
| (41) | Cheston et al. (2022) - ToDS | Items were derived from UK dementia leaflets/National Health Service website materials and mapped to Kitwood principles and DSM-5 cognitive domains. | No formal quantitative expert CVI was reported. | EFA supported a 13-item single-factor structure; variance explained=49.5%. | Cronbach alpha=0.911 in Study 1 and 0.913 in Study 2. | Differences between Black, Asian, and minority ethnic and White participants were examined, but formal cross-cultural validity or invariance was not tested. | 4-week ICC=0.74 (n=91). | Not assessed. | Not assessed. | Supported by correlations with FADS and ageism-related separation; no significant association with HADS-A, supporting discriminant validity. | Not assessed. |
| (6) | French et al. (2012) - original FADS | Themes generated through brainstorming with geropsychologists, healthcare providers and older adults. | No quantitative CVI; based on theory and previous single-item work. | PCA supported 3 factors: general fear, physical symptoms and catastrophic attitude; cumulative variance=57.1%. | Total Cronbach alpha=0.94; subscale alpha=0.80-0.94. | Not assessed in the original sample. | Not reported. | Not assessed. | Not assessed. | Supported by significant associations with STAI-Y total and trait anxiety, while state anxiety was not associated. | Not assessed. |
| (8) | Farina et al. (2023) - FAM validation version | Revised from the preliminary FAM, with wording modifications such as memory lapses. | No independent quantitative CVI reported. | EFA identified 2 factors (fear and avoidance; 18 items), confirmed by CFA in an independent international sample. | Cronbach alpha=0.80-0.90 in US and international samples. | Tested in US and international English-speaking samples; formal measurement invariance not reported. | Not reported. | Not assessed. | Not assessed. | Fear and avoidance scores correlated with memory failures, sleep disturbance, social function and quality of life as expected. | Not assessed. |
| (66) | Moon et al. (2014) - K-FADS | Translated using bilingual forward/back translation, expert committee review and pilot testing with 10 participants. | Reviewed by 4 neurologists and 2 neuropsychologists, but quantitative CVI not reported. | CFA supported the original 3-factor structure, although three items had factor loadings <0.60 and were retained. | Total Cronbach alpha=0.96; subscale alpha=0.93-0.96. | Rigorous translation/back-translation and cultural adaptation for Korean older adults; formal invariance not reported. | 3-month ICC=0.98 (n=20). | Not assessed. | Not assessed. | Supported by significant correlation with K-STAI trait anxiety and no association with state anxiety. | Not assessed. |
| (16) | Xu et al. (2021) - Chinese FODS | Translated with author permission using the Brislin model and expert consultation. | 7 experts; S-CVI=0.934; I-CVI=0.857-1.000. | EFA identified 4 factors; cumulative variance=77.46%. | Total Cronbach alpha=0.893; factor alpha >=0.903. | Expert consultation and pilot testing were used for Chinese cultural adaptation; formal measurement invariance not reported. | 2-week test-retest r=0.854 (n=20). | Not assessed. | Not assessed. | Item discrimination supported by CR values of 5.154-17.290. | Not assessed. |
| (18) | Deng et al. (2025) - Chinese FAM | Translated from the original FAM using Brislin forward/back translation; original authors reviewed the translation and pilot testing was conducted. | 19 experts; S-CVI=0.96; I-CVI=0.89-1.00. | EFA identified 2 factors (fear and avoidance); cumulative variance=69.08%; CFA fit was acceptable/moderate (RMSEA=0.083). | Total Cronbach alpha=0.934; fear alpha=0.939; avoidance alpha=0.915; split-half reliability=0.852. | 19 experts supported cultural adaptation and 10 content modifications; formal invariance not reported. | 4-week test-retest r=0.971 (n=30). | Not assessed. | Correlations with FODS, GAI and GDS were reported; best interpreted as convergent validity. | Item discrimination supported by CR values of 6.028-21.225. | Not assessed. |
| (28) | Zhang et al. (2022) - Chinese FADS | Translated using the Brislin model with original-author discussion and pretesting. | 6 experts; S-CVI=0.94; I-CVI=0.82-1.00. | EFA identified 3 factors: general fear, physical symptoms and cognitive/catastrophic attitude; cumulative variance=69.58%. | Total Cronbach alpha=0.934; split-half reliability=0.750. | 6 experts conducted cultural adaptation and revised ambiguous items; formal measurement invariance not reported. | Method section mentioned test-retest reliability, but specific results were not reported. | Not assessed. | Not assessed. | Item-total correlations=0.337-0.765; CR values=4.321-14.625. | Not assessed. |

Abbreviations: AD, Alzheimer disease; ADI, Anticipatory Dementia Instrument; ADRD, Alzheimer disease and related dementias; CFA, confirmatory factor analysis; CR, critical ratio; CVI, content validity index; DWS/MDWS, Dementia Worry Scale/Modified Dementia Worry Scale; EFA, exploratory factor analysis; FADS, Fear of Alzheimer's Disease Scale; FAM, Fear and Avoidance of Memory Loss scale; FOD, Fear of Dementia; FODS, Fear of Dementia Scale; GAI, Geriatric Anxiety Inventory; GDS, Geriatric Depression Scale; HADS-A, Hospital Anxiety and Depression Scale-Anxiety; ICC, intraclass correlation coefficient; I-CVI, item-level content validity index; K-FADS, Korean version of FADS; MIC, minimal important change; NRS, numerical rating scale; PCA, principal component analysis; PROM, patient-reported outcome measure; Ref., reference number in the main reference list; S-CVI, scale-level content validity index; SDC, smallest detectable change; SEM, standard error of measurement; STAI, State-Trait Anxiety Inventory; ToDS, Threat of Dementia Scale.

Note. “Not assessed” indicates that the corresponding COSMIN measurement property was not evaluated or was not extractable from the available information. Because no accepted gold standard exists for dementia fear or dementia worry, correlations with related constructs were summarized under hypothesis testing/construct validity when more appropriate.
