# Supplementary material for: Fear of dementia in adults without diagnosed dementia: an updated systematic review of prevalence, measurement, and correlates
Source: Front Public Health. 2026 Aug 28;14:1930480. doi: 10.3389/fpubh.2026.1930480 (PMC13561815; doi:10.3389/fpubh.2026.1930480)
Supplement: Supplementary file 5 [file Table_5.DOCX]

Supplementary Table 5. Detailed psychometric properties of dementia fear, dementia worry, and fear-of-memory-loss instruments

This supplementary table summarizes the reported psychometric properties of instruments measuring dementia fear, dementia worry, dementia-related anxiety, threat of dementia, and fear/avoidance of memory loss. It reports numerical reliability and validity findings; COSMIN methodological quality judgments are reported separately in Supplementary Table 4.

Panel A. Instrument characteristics and reliability

| **Ref.** | **Instrument/version** | **Source study; country/region** | **Target population** | **Items/dimensions** | **Internal consistency and test-retest reliability** |
| --- | --- | --- | --- | --- | --- |
| (6) | **FADS (original)** | French et al., 2012; United States | Community-dwelling older adults | 30 items; 3 dimensions: general fear, physical symptoms, catastrophic attitude | Total Cronbach Cronbach alpha = 0.94; subscale Cronbach Cronbach alpha = 0.94, 0.85, 0.80. Test-retest reliability NR. |
| (66) | **K-FADS** | Moon et al., 2014; Korea | Healthy older volunteers | 30 items; 3 dimensions consistent with original FADS | Total Cronbach Cronbach alpha = 0.96; subscale Cronbach Cronbach alpha = 0.93-0.96. 3-month ICC = 0.98 (n = 20). |
| (28) | **Chinese FADS** | Zhang et al., 2022; China | Community-dwelling older adults | 30 items; 3 factors: general fear, physical manifestations, cognitive attitude | Total Cronbach Cronbach alpha = 0.934; split-half reliability = 0.750. Test-retest mentioned, but coefficient NR. |
| (30) | **Chinese FADS** | Lin et al., 2025; Hong Kong, China | Community-dwelling middle-aged and older adults | 30 items; 3 dimensions consistent with original FADS | Total Cronbach Cronbach alpha = 0.912; subscale Cronbach Cronbach alpha = 0.929-0.969. 2-week ICC = 0.793 (n = 32). |
| (40) | **FODS (original)** | Lee and Jung, 2020; Korea | Community-dwelling older adults | 19 items; 3 dimensions and 5 factors | Total Cronbach Cronbach alpha = 0.927; subscale Cronbach Cronbach alpha = 0.849-0.923; Rasch separation reliability reported as good. Test-retest reliability NR. |
| (16) | **Chinese FODS** | Xu et al., 2021; China | Community-dwelling older adults | 18 items; 4 factors | Total Cronbach Cronbach alpha = 0.893; factor-level alpha reported as acceptable/good. 2-week r = 0.854 (n = 20). |
| (15) | **DWS/MDWS** | Roberts and Maxfield, 2021; United States | General adults | 12 items; single factor | Cronbach Cronbach alpha = 0.95-0.96. 4-week r = 0.90; 8-week r = 0.78. |
| (42) | **FOD scale** | Page et al., 2019; United States | Cognitively intact middle-aged and older adults | Generated from a 39-item pool; 3 factors: burden/loss, quality of life, perceived social/cognitive loss | Total Cronbach Cronbach alpha = 0.98; subscale Cronbach Cronbach alpha = 0.86-0.97. Two-year follow-up available, but test-retest not calculated because factor structure changed. |
| (53) | **ADI** | Rahman, 2016; United States | Community-dwelling middle-aged/older adults | 50 items; 6 components based on Health Belief Model | Total Cronbach Cronbach alpha = 0.896. Test-retest reliability NR. |
| (56) | **FAM (initial version)** | Farina et al., 2022; international/English-speaking sample | Older adults | 23 items; 3 factors: fear-avoidance, problematic beliefs, emotional resilience | Total Cronbach Cronbach alpha = 0.82; factor 1 Cronbach alpha = 0.87; other factor Cronbach alpha values lower (0.47 and 0.52). Test-retest reliability NR. |
| (8) | **FAM (validated version)** | Farina et al., 2023; US/international English-speaking samples | Community-dwelling older adults | 18 items; 2 factors: fear and avoidance | Subscale alpha values = 0.80-0.90 across samples. Test-retest reliability NR. |
| (18) | **Chinese FAM** | Deng et al., 2025; China | Community-dwelling older adults | 18 items; 2 factors: fear and avoidance | Total Cronbach Cronbach alpha = 0.934; factor Cronbach Cronbach alpha = 0.939 and 0.915; split-half reliability = 0.852. 4-week r = 0.971 (n = 30). |
| (41) | **ToDS** | Cheston et al., 2022; United Kingdom | Adults and university students | 13 items; single factor | Cronbach Cronbach alpha = 0.911 and 0.913 across two studies. 4-week ICC = 0.74 (95% CI 0.58-0.83; n = 91). |

Panel B. Validity evidence and rarely reported properties

| **Ref.** | **Instrument/version** | **Content validity/cultural adaptation** | **Structural validity** | **Criterion, convergent, or known-groups validity** | **Measurement error/responsiveness** |
| --- | --- | --- | --- | --- | --- |
| (6) | **FADS (original)** | Items generated through brainstorming with older-adult psychology/health professionals and older adults; quantitative CVI NR. | PCA supported a 3-factor solution; cumulative variance = 57.10%. | Associated with STAI-Y total score (r = 0.216) and trait anxiety (r = 0.287); state anxiety not significant. | NR |
| (66) | **K-FADS** | Forward/back translation; expert committee review; pilot testing with 10 volunteers. | Factor analysis/CFA supported the original 3-factor structure. | Correlated with K-STAI trait anxiety (r = 0.326); not correlated with state anxiety. | NR |
| (28) | **Chinese FADS** | Brislin translation; 6 experts; S-CVI = 0.94; I-CVI = 0.82-1.00. | EFA supported 3 factors; cumulative variance = 69.58%. | Item discrimination supported: CR = 4.321-14.625; item-total correlations = 0.337-0.765. | NR |
| (30) | **Chinese FADS** | Brislin translation; expert review; cognitive interviews with 5 lay participants; S-CVI = 0.833; I-CVI = 0.80-1.00. | EFA supported 3 factors; explained variance = 75.35%. | Convergent validity supported by perceived susceptibility/severity; known-groups validity supported by dementia-contact history. | NR |
| (40) | **FODS (original)** | Developed from fear expectation theory, literature review and concept analysis; 12 experts conducted two rounds of content validation; specific CVI NR. | EFA identified 5 factors within 3 dimensions; cumulative variance = 78.44%; Rasch analysis examined item difficulty/category functioning. | Correlated with K-FADS (r = 0.794) and NRS (r = 0.408); known-groups validity supported by fear/no-fear groups. | NR |
| (16) | **Chinese FODS** | Brislin translation after permission from original author; 7 experts; S-CVI = 0.934; I-CVI = 0.857-1.000. | EFA identified 4 factors; cumulative variance = 77.46%. | Item discrimination supported: CR = 5.154-17.290. | NR |
| (15) | **DWS/MDWS** | Modified terminology from dementia to ADRD; quantitative CVI NR. | One-factor structure supported across two studies; explained variance = 66.04%-66.70%. | Correlated with fear of dementia, subjective memory decline, generalized anxiety, health anxiety and neuroticism. | NR |
| (42) | **FOD scale** | Based on clinical experience, brainstorming and qualitative literature; quantitative CVI NR. | PCA identified 3 factors; explained variance = 25.3%. | Associated with aging anxiety and neuroticism; negatively associated with conscientiousness. | NR |
| (53) | **ADI** | Developed using HBM framework; quantitative CVI NR. | PCA with parallel analysis identified 6 components; cumulative variance = 52.48%. | Correlated with FADS (r = 0.697), subjective memory complaints, trait anxiety and depressive symptoms. | NR |
| (56) | **FAM (initial version)** | Generated from review of chronic illness fear scales, professional/patient interviews and older-adult pilot testing; quantitative CVI NR. | EFA/PCA identified 3 factors; explained variance >50%. | Correlated positively with geriatric anxiety (r = 0.47) and memory failures (r = 0.47), and negatively with quality of life. | NR |
| (8) | **FAM (validated version)** | Based on initial FAM with wording refinements; independent quantitative content validity NR. | EFA identified a 2-factor structure; CFA supported the structure in an independent international sample. | Fear/avoidance scores associated with FADS, GAI, GDS, memory failures, sleep problems, social functioning, verbal memory and quality of life. | NR |
| (18) | **Chinese FAM** | Brislin forward/back translation; original author review; 19 experts; S-CVI = 0.96; I-CVI = 0.89-1.00; 10 content adaptations. | EFA supported 2 factors; cumulative variance = 69.08%; CFA fit acceptable (RMSEA = 0.083). | Correlated with FODS (r = 0.596), GAI (r = 0.388) and GDS (r = 0.469); item discrimination CR = 6.028-21.225. | NR |
| (41) | **ToDS** | Items derived from UK public dementia information and mapped to Kitwood/personhood and DSM-5 cognitive domains; quantitative expert CVI NR. | EFA supported a single-factor solution; explained variance = 49.47%-49.5%. | Associated with FADS total score (r = 0.204), positively with FADS catastrophic attitude, negatively with ageism/separation, and not associated with HADS anxiety. | NR |

Abbreviations: ADI, Anticipatory Dementia Instrument; ADRD, Alzheimer disease and related dementias; CFA, confirmatory factor analysis; CR, critical ratio; CVI, content validity index; DWS, Dementia Worry Scale; EFA, exploratory factor analysis; FADS, Fear of Alzheimer's Disease Scale; FAM, Fear and Avoidance of Memory Loss scale; FOD, Fear of Dementia; FODS, Fear of Dementia Scale; GAI, Geriatric Anxiety Inventory; GDS, Geriatric Depression Scale; HADS, Hospital Anxiety and Depression Scale; HBM, Health Belief Model; ICC, intraclass correlation coefficient; I-CVI, item-level content validity index; K-FADS, Korean version of FADS; MDWS, Modified Dementia Worry Scale; NR, not reported or not extractable; NRS, numerical rating scale; PCA, principal component analysis; PROM, patient-reported outcome measure; Ref., reference number in the main reference list; S-CVI, scale-level content validity index; STAI, State-Trait Anxiety Inventory; ToDS, Threat of Dementia Scale.

Note. NR indicates that the psychometric property was not reported or could not be extracted from the available materials. This table presents reported psychometric results and should be read alongside Supplementary Table 4, which reports the COSMIN methodological quality judgments.
